# Genome-wide QTL analysis of tomato fruit cuticle deposition and composition

**DOI:** 10.1038/s41438-021-00548-5

**Published:** 2021-05-01

**Authors:** Rida Barraj Barraj, Patricia Segado, Rocío Moreno-González, Antonio Heredia, Rafael Fernández-Muñoz, Eva Domínguez

**Affiliations:** 1 Instituto de Hortofruticultura Subtropical y Mediterránea La Mayora, Universidad de Málaga—Consejo Superior de Investigaciones Científicas, Departamento de Mejora Genética y Biotecnología, Estación Experimental La Mayora, Algarrobo-Costa, E-29750 Málaga, Spain; 2 Instituto de Hortofruticultura Subtropical y Mediterránea La Mayora, Universidad de Málaga—Consejo Superior de Investigaciones Científicas, Departamento de Biología Molecular y Bioquímica, Universidad de Málaga, E-29071 Málaga, Spain; 3 Departament de Bioquímica i Fisiologia and Institut de Recerca en Nutrició i Seguretat Alimentària (INSA-UB), Universitat de Barcelona (UB), E-08028 Barcelona, Spain

**Keywords:** Plant breeding, Natural variation in plants

## Abstract

Genetics of traits related to fruit cuticle deposition and composition was studied in two red-fruited tomato species. Two mapping populations derived from the cross between the cultivated tomato (*Solanum lycopersicum* L.) and its closest relative wild species *Solanum pimpinellifolium* L. were employed to conduct a QTL analysis. A combination of fruit cuticle deposition, components and anatomical traits were investigated and the individual effect of each QTL evaluated. A total of 70 QTLs were identified, indicating that all the cuticle traits analyzed have a complex polygenic nature. A combination of additive and epistatic interactions was observed for all the traits, with positive contribution of both parental lines to most of them. Colocalization of QTLs for various traits uncovered novel genomic regions producing extensive changes in the cuticle. Cuticle density emerges as an important trait since it can modulate cuticle thickness and invagination thus providing a strategy for sustaining mechanical strength without compromising palatability. Two genomic regions, located in chromosomes 1 and 12, are responsible for the negative interaction between cuticle waxes and phenolics identified in tomato fruit. Several candidate genes, including transcription factors and structural genes, are postulated and their expression analyzed throughout development.

## Introduction

The plant cuticle, as the interface between the plant and the environment, plays a role in plant performance, fruit quality and postharvest^1,2^. Among the functions of agronomic relevance are the protection from water loss and UV radiation, its function as a thermal regulator, and the mechanical protection against pathogens and abiotic stresses, either environmental or derived from internal tissues^3^. Thus, the cuticle participates in the protection from fungal penetration, fruit cracking, organ dehydration and fusion, and in controlling organ growth. The cuticle is composed of a lipid matrix of esterified polyhydroxy fatty acids named cutin, polysaccharides from the cell wall, phenolic compounds and waxes, a mixture of soluble lipids and triterpenoids that can be located within the cuticle matrix or deposited on the surface^3^. Waxes play a critical role in the control of water loss from internal tissues and can also confer some mechanical strength^4,5^. In tomato fruit cuticle, waxes play a small part in the mechanical resistance, being the phenolic fraction the main modulator of the cuticle’s stiffness, deformation, and strength^6,7^. This phenolic domain is composed of cinnamic acids derivatives present during fruit growth and increases significantly during ripening with the incorporation of the flavonoid chalconaringenin, responsible for the yellow-orange color of the cuticle in red ripe tomatoes^6^.

The cuticle is mainly located to the outer epidermal cell wall, and depending on the species, can cutinize the epidermal anticlinal walls and even collenchyma walls. In crops such as apple^8^, mango^9^, cucumber^10^, and tomato^11^, among others, various degrees of fruit cuticle invagination have been observed. A relation between cuticle thickness and invagination and the biomechanical properties of the cuticle has been established^11^. Moreover, a relation between cuticle thickness and invagination, skin strength and fruit cracking resistance has been indicated^12,13^. However, these two traits, cuticle thickness and invagination have a negative impact on the sensory properties of the skin, which are especially relevant in small fruits that are consumed unpeeled^14^. On the other hand, a thick cuticle that encapsulates the epidermal and some collenchyma cells would also have a negative impact on fruit peeling for processing and canning since it would difficult the removal of the skin and partially eliminate the collenchyma, a tissue that contributes to the mechanical properties of the fruit^11,15,16^.

Understanding the genetic basis of cuticle development and composition is needed for breeding in crop species. Although numerous genes related to cuticle biosynthesis have been identified, most of these have been uncovered after the analysis of mutagenized populations and/or gene silencing. However, their participation in the genetic variation present in natural populations, cultivars and wild species closely related to crops is uncertain. Natural variation is a valuable source of beneficial traits for breeding. Limited work based on variation in natural plant populations has been carried out to study the cuticle, and mainly focused on waxes^17–21^. Analysis of genes involved in anatomical and biophysical traits of the cuticle, many of great importance for plant performance and fruit quality, are scarce at most. In this sense it should be mentioned the relationship found between postharvest water loss in pepper and differences in wax composition and cutin amount^22^ and the identification of quantitative trait loci (QTL) for cuticle thickness and invagination in tomato and cucumber^10,19^.

Tomato genetics and breeding heavily relies on exploiting natural variation from related wild species. Wild tomato species have been reported to exhibit cuticle diversity for some anatomical and chemical traits^18^, however, its genetics has only been studied in lines derived from the green-fruited *Solanum pennellii* Dun.^19^. In this work, we have explored for the first time the cuticle variability of red-fruited species in two populations derived from the interspecific cross between the domesticated tomato (*S. lycopersicum* L.) and its closest wild relative *S. pimpinellifolium* L. A QTL analysis of the different cuticle components and anatomical traits was performed revealing a complex polygenic nature that combined additive and epistatic interactions among the different QTLs.

## Results

### Analysis of the parental lines

**Fig. 1:**
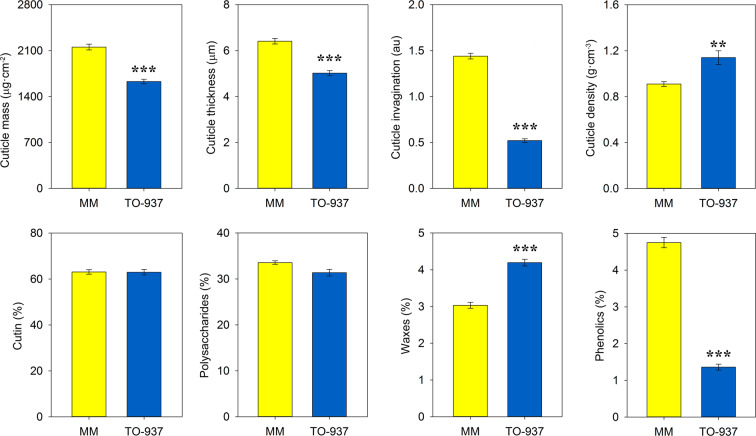
Comparison between the parental lines MM and TO-937 for the different cuticle traits studied. Red ripe fruits were analyzed. Data are presented as means ± SE. Asterisks indicate significant differences between both lines. Arbitrary units (au)

Cuticle analysis of the cultivated tomato “Moneymaker” (MM) and the wild accession TO-937 showed significant differences for most of the traits studied (Fig. 1). Thus, red ripe cuticles of TO-937 displayed a significantly lower cuticle mass, thickness and invagination. However, the decrease in thickness and invagination was not proportional to the reduction of cuticle mass, causing a significant increase in cuticle density compared to MM. The percentage of total waxes present in the cuticle of TO-937 was statistically higher compared to MM whereas phenolics were significantly lower. No significant differences in the percentages of the major cuticle components, cutin and polysaccharides, were detected.

**Fig. 2:**
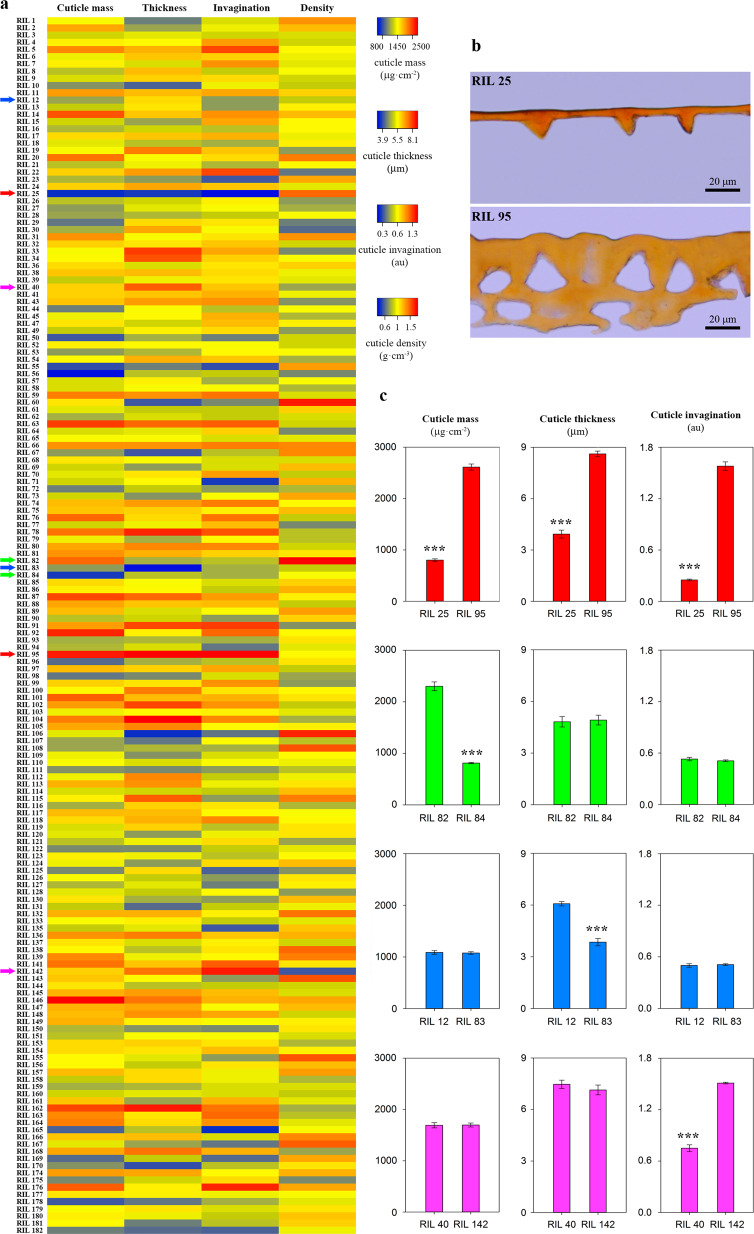
Interaction among the different traits related to cuticle deposition. **a** Heat map of the RIL population for cuticle mass, thickness, invagination and density. Arrows indicate the lines further shown in detail. **b** Cross-sections of the isolated cuticle of two RILs stained with Sudan IV. **c** Pair-wise comparisons of RILs with specific combinations of cuticle mass, thickness and invagination. Asterisks indicate significant differences with *p* < 0.001. Recombinant inbred line (RIL), arbitrary units (au)

Cuticle characterization of the RIL (recombinant inbred line) population showed transgressive inheritance for most of the traits analyzed, with lines displaying values significantly higher and lower than the parental lines. This indicates that both parental lines have positive QTLs for the traits analyzed. For example, RILs with cuticle masses significantly lower than TO-937 (100 lines) and higher than MM (14 lines) were identified. Similarly, despite the very low percentage of cuticle phenolics detected in TO-937, 21 RILs showed values significantly higher than MM, indicating a positive contribution of TO-937 alleles. Cuticle mass was deconstructed into three parameters: thickness, degree of invagination and density. The combination of which provides a good approximation to the overall process of cuticle deposition. Results of the RIL population for these traits are shown in Fig. 2a. Although increasing cuticle masses could be expected to translate into thicker and more invaginated cuticles (Fig. 2b, c), this is not always the case (Fig. 2a). Comparison of RIL 82 and RIL 84 shows that significantly different cuticle masses can display similar thickness and invagination (Fig. 2c). At the same time, significant differences in cuticle thickness can be observed in lines with similar cuticle mass and invagination (RIL 12 vs. RIL 83), as well as significant changes in the degree of invagination without affecting cuticle mass or thickness (RIL 40 vs. RIL 142).

### Cuticle mass

**Fig. 3:**
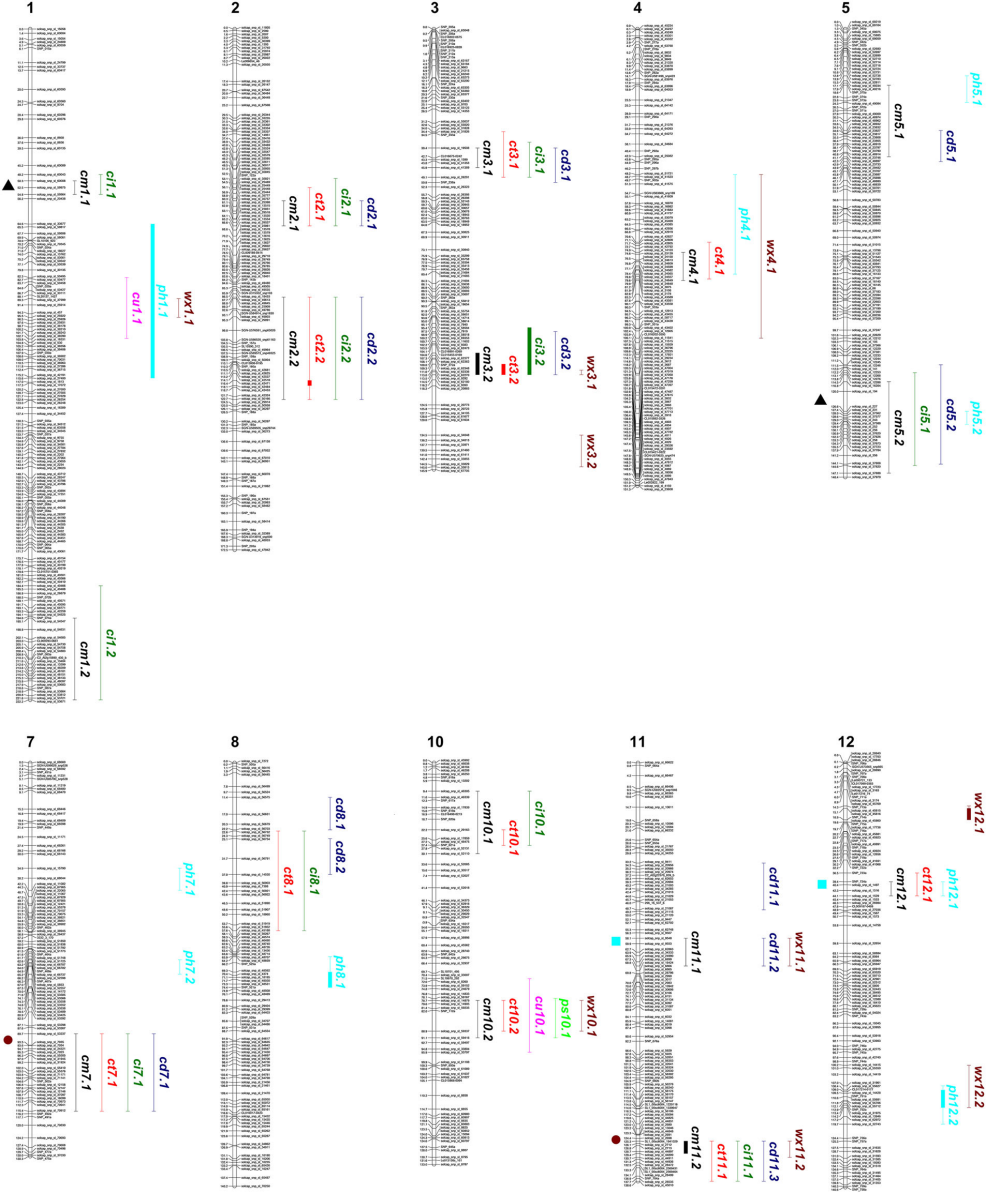
Map of tomato chromosomes showing QTL regions identified in the RIL and IL populations for the different cuticle traits. Cuticle mass (cm), cuticle thickness (ct), cuticle invagination (ci), cuticle density (cd), percentage of cutin (cu), percentage of polysaccharides (ps), percentage of phenolics (ph), and percentage of waxes (wx). Bold lines represent QTL regions with *p* < 0.001. Symbols with the same shape and color located to the left of the chromosomes denote regions with epistatic interactions. Symbol color indicates the trait. Numbers to the left of chromosomes indicate genetic distances expressed in cM (Kosambi mapping function). Recombinant inbred line (RIL), inbred line (IL)

Seventy QTLs were detected for different cuticle traits within both populations (Fig. 3). Statistically significant QTLs in the RIL population represent 34 of the total detected. The rest of the QTLs were identified as statistically significant in the IL population and most of them coincided with genomic regions that displayed non-statistically significant QTLs in the RIL population, that is, with probability below the 0.99 threshold. In these instances, the peak marker observed in the RIL population is given in the corresponding Tables. Out of the 15 QTLs identified for cuticle mass (cm) (Table 1), 13 had lyc effect that is, the MM allelic region increased cuticle mass whereas two other QTLs, *cm5.2* and *cm10.2*, had pim effect, TO-937 alleles increased the cuticle mass. QTL epistatic analysis within the RIL population identified a significant interaction between *cm1.1* and *cm5.2* (LOD 5.18, 15% variance explained) with pim effect. This indicated that the lyc allelic *cm1.1* region increases the pim effect of *cm5.2*.

**Table 1:** QTLs identified in the RIL and IL populations for cuticle mass

QTL	Effect	LOD	PVE	*P*	Peak marker	Population
*cm1.1*	lyc	–	–	–	solcap59975	IL
*cm1.2*	lyc	–	–	–	solcap48181	IL
*cm2.1*	lyc	2.8	6.9	0.989	solcap23851	RIL/IL
*cm2.2*	lyc	3.67	9.8	0.997	solcap42471	RIL/IL
*cm3.1*	lyc	4.65	11.9	1.000	solcap41353	RIL/IL
*cm3.2*	lyc	6.85	17.0	1.000	solcap62180	RIL/IL
*cm4.1*	lyc	3.61	7.8	0.998	solcap3108	RIL/IL
*cm5.1*	lyc	3.04	6.8	0.992	solcap23797	RIL/IL
*cm5.2*	pim	3.66	8.9	0.998	solcap37808	RIL/IL
*cm7.1*	lyc	–	–	–	–	IL
*cm10.1*	lyc	3.94	8.1	0.999	solcap46305	RIL/IL
*cm10.2*	pim	3.08	7.3	0.995	solcap14879	RIL/IL
*cm11.1*	lyc	–	–	–	–	IL
*cm11.2*	lyc	6.02	13.8	1.000	solcap100974	RIL/IL
*cm12.1*	lyc	3.84	9.4	0.998	solcap1497	RIL/IL

QTLs are named with a trait abbreviation followed by chromosome number and the number of the QTL within the chromosome. Effect refers to the allele (lyc/pim) that increases the trait. lyc, *S. lycopersicum* MM. pim, *S. pimpinellifolium* TO-937

*PVE* percentage of variance explained by the QTL in the RIL population, *P* probability. Marker closest to the maximum LOD (logarithm of the odds) score is also shown. *cm* cuticle mass, *RIL* recombinant inbred line, *IL* inbred line

**Fig. 4:**
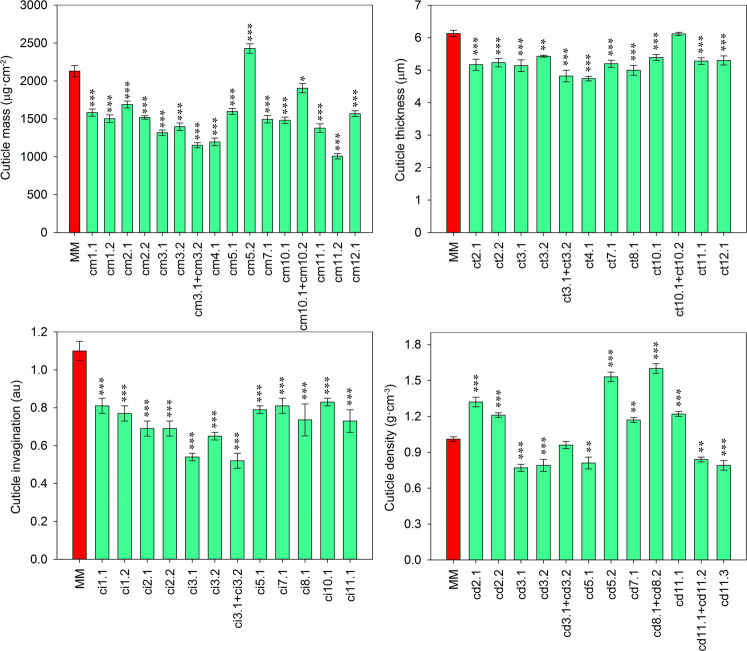
Comparison between the parental MM and the ILs harboring QTLs related to cuticle deposition. Red ripe fruits were analyzed. Data are presented as means ± SE. Asterisks indicate significant differences with MM (*p < 0.05; **p < 0.01; ***p < 0.001). Inbred line (IL), cuticle mass (cm), cuticle thickness (ct), degree of cuticle invagination (ci), cuticle density (cd), and arbitrary units (au)

To study the individualized effect of *cm3.1* and *cm3.2*, subILs were generated from the IL sp3-2 that separated and shortened these QTLs (Supplementary Fig. 1). The highest effect on cuticle mass was observed in the IL harboring *cm11.2* with a 2-fold reduction compared to MM, followed by *cm4.1* and the combination *cm3.1* + *cm3.2* (Fig. 4). Each of the QTLs of chromosome 3 showed a similar reduction in cuticle, ∼38%, whereas the combination showed a small additive effect, reducing 45% the cuticle mass. On the other hand, the pim effect of *cm5.2* produced an increased 12–14% of cuticle mass. Although *cm10.2* was not separated from *cm10.1*, comparison of *cm10.1* + *cm10.2* with *cm10.1* showed an increase in cuticle mass to levels almost similar to those of MM, indicating that the pim effect of *cm10.2* almost counteracted the lyc effect of *cm10.1*. In order to shorten *cm11.1*, overlapping subILs were generated from the sp11-1 line (Supplementary Fig. 2). Despite the significant decrease in cuticle mass observed in sp11-1 compared to MM, all the derived subILs were similar to MM, indicating that *cm11.1* must be located to a lyc region present in all the subILs except sp11-1 (Supplementary Fig. 2).

Of the 11 QTLs identified for cuticle thickness (ct) (Supplementary Table 1) the biggest decrease, ∼20%, was observed for *ct4.1* and the combination of the two QTLs of chromosome 3 *ct3.1* + *ct3.2* (Fig. 4). Again, the combination *ct3.1* + *ct3.2* showed additive effect compared to the individual QTLs. With the exception of *ct10.1* + *ct10.2*, all the lines harboring the individual or double QTLs showed a reduction in cuticle thickness compared to MM (Fig. 4). Comparison of the line harboring *ct10.1* + *ct10.2* with *ct10.1* showed an increase in cuticle thickness to levels similar to MM, thus supporting the pim effect of *ct10.2* observed in the RIL population. All the QTLs identified for cuticle invagination (ci) showed lyc effect (Supplementary Table 2) as it can be inferred from the significant decrease observed in comparison to MM (Fig. 4). The highest reduction in invagination, around 50%, was observed for *ci3.1* and the combination *ci3.1* + *ci3.2*, indicating that, contrary to what was observed for cuticle mass and thickness, *ci3.1* had a dominant effect on the trait.

Regarding cuticle density (cd), QTLs with both lyc and pim effect were observed (Supplementary Table 3). The lowest decrease in density was observed in the ILs with *cd3.1*, *cd3.2* and *cd5.1* whereas those containing *cd5.2* and *cd8.2* showed the highest increase compared to MM (Fig. 4). Accumulation of *cd3.1* + *cd3.2* counteracted the effect on density of the individual QTLs and the combination was similar to MM. Despite *cd8.1* was the only statistically significant QTL identified in the RIL population (Supplementary Table 3), its individualized effect could not be studied in the IL population since none of the lines carried the most proximal region of chromosome 8 as TO-937. However, since *cd8.1* had lyc effect and *cd8.2* pim, it is possible that the effect observed for *cd8.2* (Fig. 4) is indeed the result of a lyc × pim interaction between both QTLs. Generation of new lines and further analyses will be necessary to understand the effect of this region. Cuticle density analysis of sp11-1 displayed a significant decrease in cuticle density whereas the derived subILs had a significantly higher density compared to MM (Supplementary Fig. 2). The decrease in density observed in sp11-1 could be the result of the lower cuticle mass associated with *cm11.1*, while the increase in cuticle density detected in the subILs could be explained as the result of a putative QTL (*cd11.1*) with pim effect located in the overlapping region present in all the subILs. Nevertheless, these two presumed QTLs need to be further individualized and their effects studied.

### Phenolics

Nine QTL regions, in chromosomes 1, 4, 5, 7, 8, and 12, were identified in the RIL and IL populations (Table 2). Despite the very low percentage of phenolics (ph) present in TO-937 cuticle, four QTLs with pim effect were identified: *ph4.1*, *ph5.1*, *ph5.2*, and *ph12.1*. A significant epistatic interaction (LOD 5.57, 10.9% variance explained) with pim effect was identified between *ph12.1* and a region in chromosome 11 (Fig. 3). This indicated that the lyc allelic region in chromosome 11 increases the pim effect of *ph12.1* and could explain why *ph12.1* was not statistically significant in the RIL population but showed marked differences in the IL population. This chromosome 11 region did not have an effect on phenolics by itself (data not shown).

**Table 2:** QTLs identified in the RIL and IL populations for the percentage of cuticle components

QTL	Effect	LOD	PVE	*P*	Peak marker	Population
*ph1.1*	lyc	11.37	26.7	1.000	solcap457	RIL/IL
*wx1.1*	pim	3.54	9.2	0.996	solcap25914	RIL
*cu1.1*	lyc	3.38	8.9	0.996	solcap25922	RIL
*wx3.1*	pim	3.13	7.4	0.992	solcap62270	RIL/IL
*wx3.2*	pim	–	–	–	solcap61411	IL
*ph4.1*	pim	–	–	–	snp302a	IL
*wx4.1*	pim	–	–	–	–	IL
*ph5.1*	pim	4.36	10.3	1.000	solcap49234	RIL
*ph5.2*	pim	2.90	7.2	0.991	solcap37562	RIL
*ph7.1*	lyc	3.39	9	0.998	solcap68044	RIL/IL
*ph7.2*	lyc	3.02	7.9	0.993	solcap5853	RIL/IL
*ph8.1*	lyc	5.64	14.3	1.000	solcap48550	RIL/IL
*wx10.1*	lyc	3.07	8	0.992	snp112a	RIL/IL
*cu10.1*	pim	–	–	–	snp112a	IL
*ps10.1*	lyc	–	–	–	solcap59337	IL
*wx11.1*	lyc	–	–	–	–	IL
*wx11.2*	pim	–	–	–	solcap100974	IL
*ph12.1*	pim	–	–	–	solcap1516	IL
*ph12.2*	lyc	5.40	13.3	1.000	solcap55706	RIL/IL
*wx12.1*	lyc	5.84	12.6	1.000	solcap45816	RIL/IL
*wx12.2*	pim	3.48	7.2	0.997	solcap55712	RIL/IL

QTLs are named with a trait abbreviation followed by chromosome number and the number of the QTL within the chromosome. Effect refers to the allele (lyc/pim) that increases the trait. lyc, *S. lycopersicum* MM. pim, *S. pimpinellifolium* TO-937

*PVE* percentage of variance explained by the QTL in the RIL population, *P* probability. Marker closest to the maximum LOD (logarithm of the odds) score is also shown. *ph* phenolics, *wx* waxes, *cu* cutin, *ps* polysaccharides, *RIL* recombinant inbred line, *IL* inbred line

**Fig. 5:**
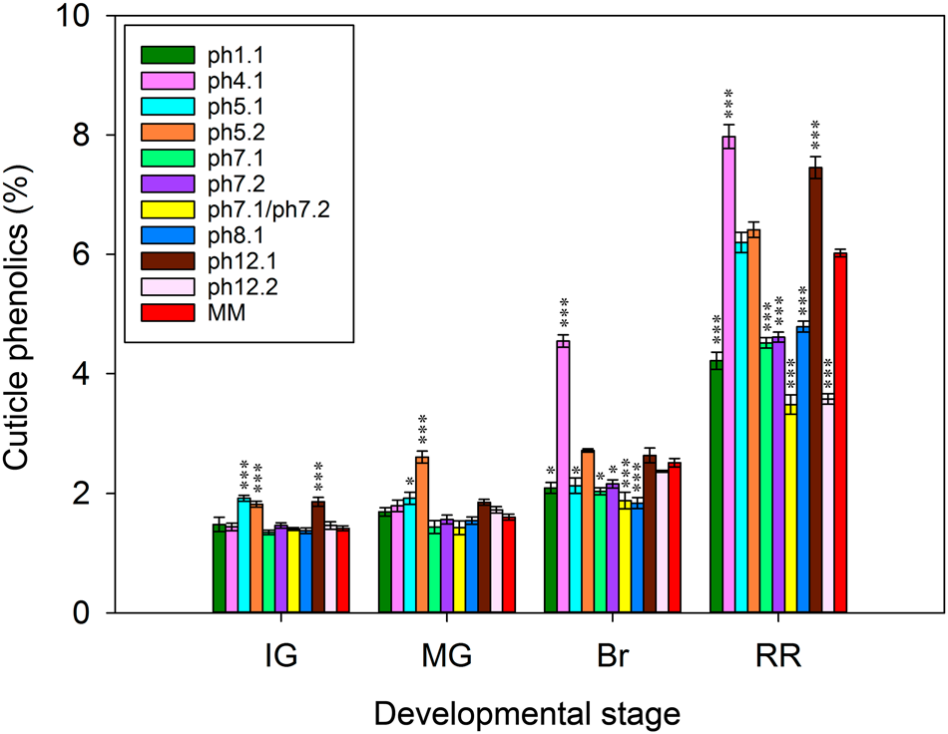
Percentage of cuticle phenolics from fruits harvested at four stages of development. The parental line MM and the ILs harboring QTL regions related to cuticle phenolics (ph) are shown. Data are presented as means ± SE. Asterisks indicate significant differences with MM (*p < 0.05; **p < 0.01; ***p < 0.001). Inbred line (IL), immature green (IG), mature green (MG), breaker (Br), and red ripe (RR)

In order to individually study *ph7.1* and *ph7.2*, subILs derived from sp7-2 were generated (Supplementary Fig. 2). The effects of each phenolic QTL were analyzed in the IL population and compared with the recurrent parental line MM at different stages of development (Fig. 5). All the lines containing the QTLs showed significant differences in the percentage of phenolics at red ripe compared to MM, with the exception of *ph5.1* and *ph5.2* that only showed a slight but non-significant increase in phenolics. The other QTLs with pim effect, *ph4.1* and *ph12.1*, did show a significant increase in the percentage of cuticle phenolics, 30 and 20% respectively, compared to MM. Regarding the QTLs with lyc effect, the biggest reduction of phenolics, ∼40%, was observed in *ph12.2* and the combined effect of *ph7.1* + *ph7.2*, whereas the remaining QTLs showed a similar reduction in the percentage of phenolics at red ripe. Surprisingly *ph1.1*, despite being the major QTL, showed an intermediate reduction in phenolics, comparable to *ph7.1*, *ph7.2*, and *ph8.1*. A noticeable additive effect was observed for the QTLs located in chromosome 7, with the combination *ph7.1* + *ph7.2* displaying a much lower percentage of phenolics than *ph7.1* or *ph7.2*. Although the tomato fruit cuticle has a marked increase in phenolics during ripening, there is a small amount of phenolic acids that accumulate during development^6^. Hence, to extend the knowledge of the effect of these QTLs, the percentage of phenolics was also studied throughout development (Fig. 5). Interestingly, at the immature and mature green stages the ILs containing *ph5.1* and *ph5.2* showed significantly higher values than MM. However, these differences disappeared during ripening. It is precisely at the onset of ripening, when the differences between most of the other lines and MM became apparent. This suggests that, except for *ph5.1* and *ph5.2*, the genes underneath these QTLs play an important role in the cuticle during ripening but not at earlier stages of development.

### Waxes, cutin, and polysaccharides

**Fig. 6:**
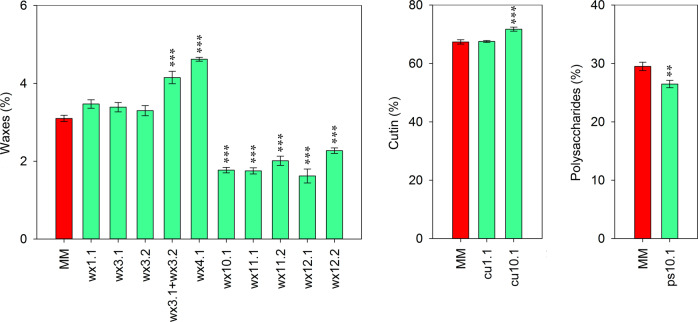
Comparison between the parental MM and the ILs with the QTLs related to the percentage of cutin, polysaccharides and waxes. Red ripe fruits were analyzed. Data are presented as means ± SE. Asterisks indicate significant differences with MM (*p < 0.05; **p < 0.01; ***p < 0.001). Inbred line (IL), cutin (cu), polysaccharides (ps), and waxes (wx)

Nine QTLs were identified for the percentage of waxes (wx), six with pim effect, and 3 with lyc effect (Table 2). A percentage of waxes significantly higher than MM was observed for the ILs containing *wx4.1* and the combination *wx3.1* + *wx3.2* (Fig. 6). However, the ILs that individualized the QTLs *wx3.1* and *wx3.2* did not show significant effects on waxes. Surprisingly, the line harboring *wx1.1* did not show any difference with MM for waxes, and the two other QTLs with pim effect, wx*11.2* and wx*12.2* showed, contrary to what it was expected, lower percentage of waxes. Finally, *wx10.1*, *wx11.1*, and *wx12.1* showed a significant decrease in waxes compared to MM. The QTL *wx11.1* was identified and located after the analysis of the subILs derived from sp11-1 (Supplementary Fig. 2), similarly to what it has already been explained for cuticle mass and density. The significant epistatic interaction (LOD 5.28, 15.7% variance explained) identified between *wx11.2* and a region in chromosome 7 (Fig. 3) could explain the decrease in waxes observed in the IL containing *wx11.2* (Fig. 6). Whereas *wx11.2* had pim effect and the chromosome 7 region lyc effect, the interaction showed an increased lyc effect. This region in chromosome 7 did not show an effect on waxes (data not shown). It is possible that a similar epistatic interaction, not detected in the QTL analysis were responsible for the change of effect detected in *wx12.2* and the absence of effect of *wx1.1*.

Despite no significant differences were detected for the percentage of cutin (cu) and polysaccharides (ps) between the parental lines, two QTLs for the percentage of cutin were identified in the RIL population, *cu1.1* with lyc effect and *cu10.1* with pim effect (Table 2). Additionally, one QTL for the percentage of polysaccharides was detected, *ps10.1*, with lyc effect that colocalized with *cu10.1*. Although a peak for percentage of polysaccharides was found in chromosome 1 in the QTL analysis, coinciding with the *cu1.1* region, it was below the statistically significant threshold. Analysis of the ILs containing the different QTLs for cutin and polysaccharides and the parental line MM is shown in Fig. 6. The lines containing *cu10.1* and *ps10.1* displayed a statistically higher and lower percentage of cutin and polysaccharides, respectively. However, the line with *cu1.1* did not show any significant difference in cutin compared with MM.

### Analysis of the genomic regions

Genes located within each QTL region were investigated in order to identify candidate genes that were reported in the literature to affect cuticle synthesis (Table 3). Of the several known tomato genes involved in cuticle deposition, none of them located within any of the QTL regions. Indeed, only a few cuticle genes were identified in the QTLs. Despite the overlap between *ph1.1* and *wx1.1*, each showed a different peak marker. *MYB12*, a transcription factor known to affect the phenolic metabolic pathway was closest to the *ph1.1* and *cu1.1* peak marker whereas *LONG CHAIN ACYL SYNTHETASE 1* (*LACS1*), a gene involved in wax and cutin biosynthesis, was closer to the *wx1.1* peak. Several *β-KETOACYL-COENZYME A SYNTHASE* (*KCS*) genes, involved in the elongation of very-long chain fatty acids, were identified in the QTLs. *ECERIFERUM6* (*CER6*) was located in the peak of *cm2.2*, *FIDDLEHEAD* (*FDH*), and *KCS11-like1*, a gene very similar to *Arabidopsis KCS11*, were located in the *ph8.1* peak marker or close to it, respectively; finally, *KCS11-like2* was located within *wx12.1*. Additionally, putative tomato orthologs of *Arabidopsis* wax genes were also identified in QTLs related to cuticle mass, although far from the peak markers.

**Table 3:** Cuticle related genes reported in the literature identified in the QTLs

QTLs	Gene symbol	Gene locus	Species	Literature
*ph1.1/wx1.1/cu1.1*	***LACS1***	Solyc01g079240	Arabidopsis	Lü et al.^41^
	***MYB12***	Solyc01g079620	Tomato	Adato et al.^38^
*ph1.1*	*DET1*	Solyc01g056340	Tomato	Davuluri et al.^40^
*cm1.2/ci1.2*	*CER9*	Solyc01g107880	Arabidopsis	Lü et al.^51^
	*LACS2*	Solyc01g109180	Arabidopsis	Schnurr et al.^52^
*cm2.2/ct2.2/ci2.2/cd2.2*	***CER6***	Solyc02g085870	Tomato	Leide et al.^26^
	*CER13*	Solyc02g086500	Arabidopsis	Rashotte et al.^53^
*ph4.1/wx4.1*	*PAS2*	Solyc04g014370	Arabidopsis	Bach et al.^54^
*cm5.1/ph5.1*	*GL1*	Solyc05g008250	Arabidopsis	Xia et al.^55^
*cm5.2/ci5.1/cd5.2*	*CER10*	Solyc05g054490	Arabidopsis	Rashotte et al.^53^
	*CYP77A6*	Solyc05g055400	Arabidopsis	Li-Beisson et al.^56^
*cm7.1/ct7.1/ci7.1/cd7.1*	*TAGL1*	Solyc07g055920	Tomato	Giménez et al.^23^
*ph8.1*	***FDH***	Solyc08g067260	Arabidopsis	Voisin et al.^27^
	*KCS11 like*	Solyc08g067410	Tomato	–
*wx12.1*	*KCS11 like2*	Solyc12g006820	Tomato	–

Genes located on the peak marker, or <5 genes away from the peak marker, are shown in bold. Underlined genes are located 10–20 genes from the peak marker. Genes in regular case are located >20 genes away from the peak marker. When more than one QTL is shown, the gene/s indicated are located within the overlapping regions

*cm* cuticle mass, *ct* cuticle thickness, *ci* cuticle invagination, *cd* cuticle density, *ph* phenolics, *cu* cutin, *wx* waxes

**Fig. 7:**
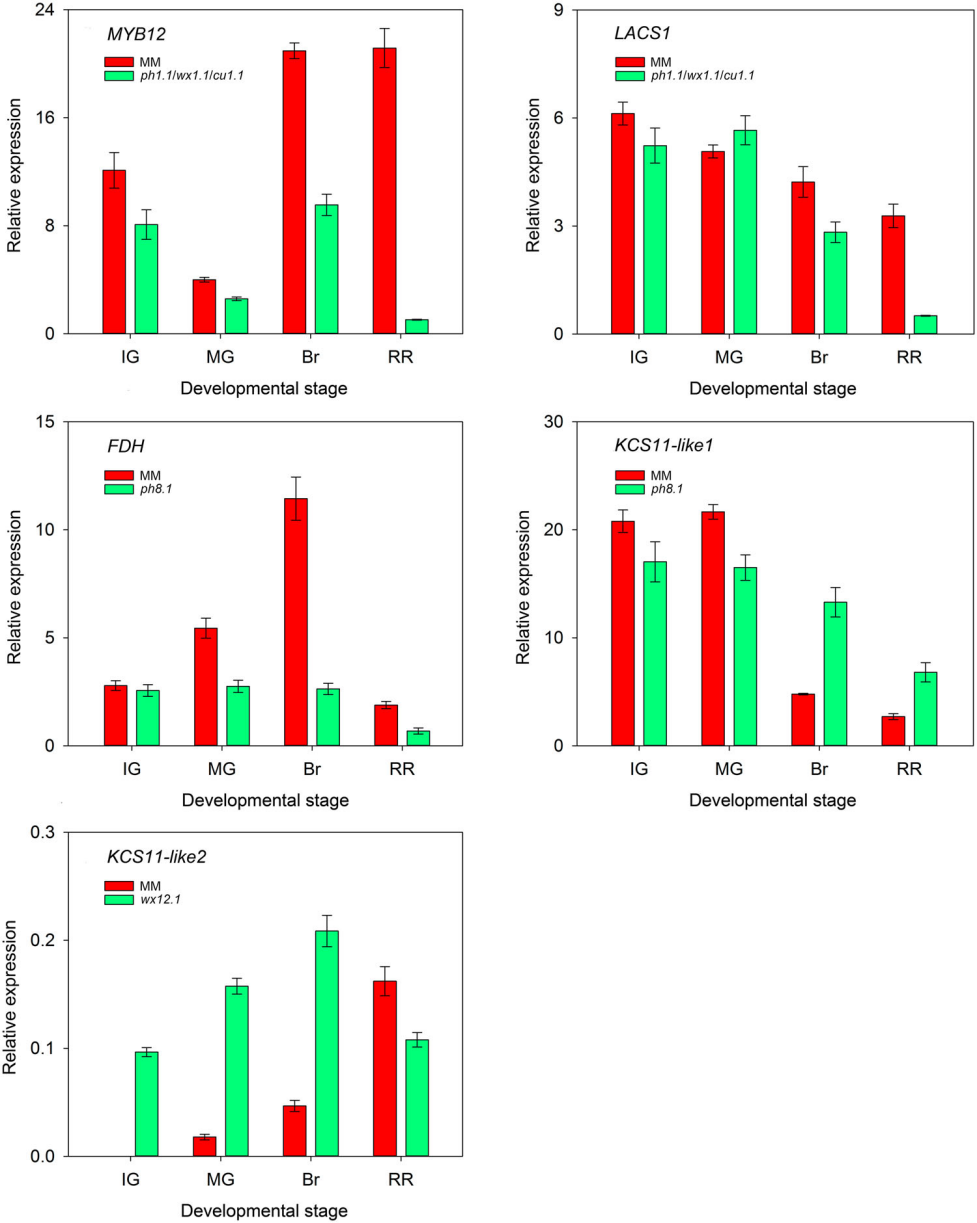
Relative expression throughout epicarp development. MM and the ILs containing the QTLs *ph1.1*/*wx1.1*/*cu1.1*, *ph8.1*, and *wx12.1* were analyzed for the putative candidate genes *MYB12*, *LACS1*, *FDH*, *KCS11-like1*, and *KCS11-like2*. Data are presented as mean ± SE. Inbred line (IL), phenolics (ph), waxes (wx), cutin (cu), immature green (IG), mature green (MG), breaker (Br), and red ripe (RR)

Comparative expression analyses of some putative candidate genes related to waxes and phenolics of MM and the corresponding ILs are shown in Fig. 7. The IL harboring *ph1.1* showed a drastic decrease of *MYB12* expression during ripening compared with MM, 2.8-fold at breaker and 32-fold at red ripe. These results are in accordance with the reduction in cuticle phenolics detected from the breaker stage until red ripe for *ph1.1* (Fig. 5). *LACS1* only showed a small decrease at breaker followed by a 8.7-fold reduction at red ripe (Fig. 7). *FDH* expression in MM increased during growth with a notable maximum at breaker whereas in the IL with *ph8.1* the expression level did not change with growth and displayed a 5-fold decrease in expression at breaker compared to MM. Again, this profile coincided with the drastic decrease in cuticle phenolics already detected at breaker in the *ph8.1* line (Fig. 5). On the other hand, the *ph8.1* line showed a small increase of *KCS11-like1* expression compared to MM during ripening. Finally, the IL containing *wx12.1* showed an increased expression of *KCS11-like2* compared to MM, especially during the whole fruit growth period until breaker. Inspection of changes in the mRNA sequences for these genes between the parental lines showed that the differences present produced conserved protein changes, with the only exception of *LACS1* with a L183 (MM) to S183 (TO-937) change.

## Discussion

### Natural variation of tomato fruit cuticle shows a combination of additive and epistatic traits

A comprehensive cuticle analysis has been carried out in two populations, RIL and IL, thus favoring the detection of additional QTLs that were not significant or present in the RIL population. Understanding the interaction of these epistatic QTLs with the genetic background is important in plant breeding for the introgression of specific traits. A combination of additive and epistatic interactions was detected for all the traits analyzed. Moreover, the transgressive inheritance found in the RIL population for most of the characters analyzed indicated the presence of QTLs with a positive contribution to the trait in both parents. Of the 25 genomic regions identified spanning 10 of the 12 chromosomes, some were specific and only affected one or two traits and others were more complex and produced global changes comprising many of the parameters studied. Uncovering the complex network of genetic interactions responsible for cuticle synthesis allows for a better understanding of its natural variability, provides an insight into the evolution of the traits and their potential for breeding purposes.

QTL analysis of a tomato IL population derived from the interspecific cross between cultivated tomato and the green-fruited species *S. pennellii* found three QTLs for the amount of specific wax and cutin components and another one for cuticle thickness^19^. With the exception of the thickness QTL, none of the other regions coincide with any of the QTLs herein identified. The thickness QTL coincided with the *ct7.1* region found here. *TOMATO AGAMOUS-LIKE1* (*TAGL1*) was postulated as one of the possible candidate genes for this trait^19^. Indeed, modification of *TAGL1* expression in tomato has been shown to lead to significant changes in cuticle mass, thickness and invagination^23^. A QTL for cuticle thickness and invagination was reported in cucumber fruit and the transcription factor *SHINE1* (*SHN1*) reported as the candidate gene^10^; however, *SHN1* is neither located within any of the QTLs herein found nor expressed in tomato fruit epicarp^24^.

Of the numerous known genes involved in phenolic synthesis and regulation only *MYB12* and *DE-ETIOLATED1* (*DET1*) were located within one of the QTLs found for cuticle phenolics. Similarly, several genes have been found to participate in cuticle deposition^25^ however, none of these genes locate within the QTLs identified for any of the cuticle traits herein studied. Several genes involved in wax accumulation in *Arabidopsis* were identified in QTLs related to cuticle deposition (Table 3). Only two genes, *LACS2* and the cytochrome P450 monooxigenase *CYP77A6*, reported to alter cutin deposition in *Arabidopsis* were located within cuticle QTLs. However, since none of these genes are located close to the QTL peak markers, their role as candidate genes needs to be further analyzed. Three *KCSs* were identified in the peak markers of QTLs unrelated to waxes, *cm2.2* and *ph8.1*. In tomato, *cer6* mutant has been shown to affect cuticle mass and reduce the synthesis of *n*-alkanes while increasing triterpenoids without altering the total amount of waxes^26^. This would be in agreement with its role as candidate gene for *cm2.2*. Of the two *KCS* located around *ph8.1* peak marker, the notable reduction of *FDH* expression during ripening in the TO-937 allele indicates that this is the most likely candidate gene. Indeed, the maximum expression at breaker coincided with the massive incorporation of phenolics to the cuticle, although waxes also show a comparatively modest increase during ripening in MM (ref. ^6^). Mutation of *FDH* in *Arabidopsis* displayed an increase in cutin and waxes^27^ that was not observed in the *ph8.1* line. This apparent unlikely role of a *KCS* in cuticle phenolics could be associated with transport. It is still unknown how phenolics are transported to the cuticle, although there are several reports of phenolics esterified to cutin and waxes^28,29^. It is plausible that they are transported already esterified to a fatty acid instead of the bonding occurring within the cuticle matrix. In this context, *FDH* could be involved in the synthesis of the fatty acid domain. Putative orthologs of *KCS11* have been postulated as candidate genes for wax accumulation in eucalypt and banana leaves^20,30^. The notably increased expression throughout development of the TO-937 allele of *KCS11-like2* and the reduction in waxes observed in the *wx12.1* line suggests that this gene could be indirectly involved in wax synthesis through the shortage of substrate available for waxes. In this regard, the ubiquitous and relatively low expression of *KCS11* in *Arabidopsis* has been suggested as an indication of a general role in the synthesis of very-long-fatty acids required for growth and development^31^.

### Cuticle density as an emerging trait in fruit quality

Decomposition of cuticle mass into three anatomical and physical traits has shown that all the QTLs for cuticle mass colocalized with QTLs related to thickness, invagination and/or density (Fig. 3), indicating that changes in cuticle mass were always accompanied by changes in any of these other traits. However, changes in cuticle deposition did not always affect all the other three parameters, allowing the identification of groups of QTLs for cuticle mass with different effects. In one group of genomic regions, *cm3.1*, *cm3.2*, and *cm11.2*, cuticle mass had a small effect on thickness and invagination causing a decrease in cuticle density, whereas in another group, *cm2.1*, *cm2.2*, and *cm7.1*, the effect on thickness and invagination was more dramatic and increased the density. Also, some genomic regions for cuticle mass had only an effect on cuticle thickness, *cm4.1*, *cm10.2*, or *cm12.1*, and others such as *cm1.1* and *cm1.2* modified cuticle invagination without changing thickness. Overall, this indicates that alteration of cuticle mass does not always translate into parallel or even proportional changes in thickness, invagination and/or density. Similarly, in cucumber, a QTL for cuticle thickness and invagination and two additional ones for invagination only were reported, pointing that thickness and invagination do not always vary together^10^. The QTLs here identified provide genetic tools to independently modify some of these traits.

Differences in cuticle density among species have been reported in the literature^32,33^. However, its evolution throughout development has only been characterized in tomato, where variation between cultivars was detected as well as changes during ripening^6^. Density can modulate the mechanical properties of the cuticle, with a high density hindering the molecular motion of the polymer chains^32^. A comparative analysis of different tree species found that cuticle density only partially explained the mechanical strength of the cuticle^33^. However, a clear and significant correlation between these two parameters was reported in a study of numerous cultivars of *Diospiros kaki*^34^. Although a reduction in cuticle density was observed after removal of polysaccharides but not waxes^32^, the role of polysaccharides derived from the primary cell wall and their association and/or chemical connection to the cutin matrix still needs further investigation to properly understand their contribution to cuticle density. Additionally, several parameters such as cutin monomer composition, the relative concentration of functional groups able to crosslink, degree of polymerization and the number of secondary hydroxyl groups engaged in polymer chain interesterification could modify cutin 3D architecture and therefore affect cuticle density. However, a structural and compositional analysis of cuticle density is still missing. The material herein identified could be a potential source for these analyses.

Changes in cuticle mass through modification of cuticle density that do not translate into thicker and more invaginated cuticles, or modulation of cuticle density could provide a strategy to maintain mechanical strength while reducing undesirable cuticle traits for palatability that could be valuable for breeding purposes. In this sense, the QTL regions in chromosome 8 that provide a dense cuticle material with low thickness and invagination without affecting cuticle mass deserves further investigation.

### Genetic interaction between cuticle phenolics and waxes

Cuticle phenolics and waxes play important biophysical roles that have great agronomic importance for fruit growth on the vine and also during postharvest^2^. However, a negative correlation between cuticle waxes and phenolics, based on the modification of the phenolic metabolic pathway, either silencing *CHALCONE SYNTHASE* (*CHS*), the first committed enzyme of the flavonoid pathway, or favoring the accumulation of anthocyanins in the fruit by expression of the transcription factors *ROSEA* and *DELILA*, has been reported^35^. The nature of this relation could have a critical impact in plant breeding since it would imply the necessity to choose between increasing mechanical resistance provided by phenolics and reduced water permeability driven by waxes. A small but significant and negative correlation was observed within the RIL population (*r* = −0.275, *P* < 0.01) between the percentages of phenolics and waxes. Analysis of the colocation of waxes and phenolics genetic determinants indicate that this relation is based on the QTLs present in chromosome 1 and 12 with overlapping regions for both compounds. However, identification of QTLs that modify either phenolics or waxes, such as *ph7.1*, *ph7.2*, *ph8.1*, and *wx12.1*, indicates that there are alternative genetic strategies to avoid a compromise between increased phenolics and waxes. Moreover, colocation of QTLs with similar effect for waxes and phenolics on chromosome 4 suggests a complex interaction between these pathways. In this sense, it has recently been reported a Kelch repeat F-box protein (SMALL AND GLOSSY LEAVES1) that negatively regulates both wax and phenylpropanoid biosynthesis in *Arabidopsis*^36,37^.

*MYB12* was identified as the candidate gene behind *ph1.1* since a notable downregulation of the pim allele was detected during ripening, coinciding with the phenotypic differences observed in the cuticle. *MYB12* is the gene responsible for the *colorless fruit epidermis* (*y*) mutation in tomato and has been reported to dramatically decrease cuticle phenolics at ripe^38^. Cuticle analysis of Ailsa Craig *y*/*y* showed a significant decrease in phenolics and cutin and increase in waxes and polysaccharides compared to the wildtype (Supplementary Table 4), which would be in agreement with the overlap of the QTLs for phenolics, waxes and cutin and the almost significant QTL for polysaccharides present in chromosome 1. Transcriptome analyses of various *y* mutant alleles have shown an increase in various enzymes related to wax biosynthesis during ripening^39^ that would support a role of *MYB12* in regulating the phenolic and fatty acid biosynthetic pathways. The reduced effect on phenolics (Fig. 5) observed for the line containing the pim allele of *MYB12*, despite *ph1.1* being the major QTL with the highest additive effect, could be the result of an interaction between *MYB12* and other region/s within this wide QTL that could be masking its individual effect. In this regard *DET1*, a regulator of light signal transduction whose downregulation increases flavonoid accumulation in tomato fruits, is the only gene with a known effect in the phenolic pathway located within *ph1.1* region^40^. Also, this lesser effect on phenolics could be behind the absence of differences for waxes and cutin observed in the line harboring the overlapping QTLs *ph1.1*, *wx1.1*, and *cu1.1* and would imply that a certain degree of phenolic reduction is needed for the effect on lipids to be significant. Additionally, an interaction between *MYB12* and *LACS1*, the gene closest to *wx1.1* peak marker, could also explain the results on waxes and cutin. Although *LACS1* expression has not been reported to be modified in the transcriptomic analysis of *y* mutants^39^, the observed downregulation of the *LACS1* pim allele during ripening indicates a potential role as candidate gene for waxes and cutin. Analysis of the *Arabidopsis LACS* family has uncovered a complex network of redundant activities, with overlapping functions of *LACS1* and *LACS2* on cutin and wax biosynthesis and *LACS1* and *LACS4* on wax biosynthesis^41,42^; hence the absence of effect on waxes and cutin could also be due to the involvement of other *LACS* genes. Individualized analysis of both genes and potential compensating roles of other *LACS* genes need to be addressed to clarify their effects.

## Materials and methods

### Plant material and subIL selection

A 169 RIL population was employed to carry out QTL analyses. This population is an F_8_ derived from the interspecific cross between *Solanum lycopersicum* L. “Moneymaker” (MM) and *Solanum pimpinellifolium* L. “TO-937” (TO-937) by single seed descent^43^. An inbred line (IL) population consisting of 52 lines derived from the recurrent backcross of TO-937 into MM background and selected for harboring specific introgressions,^44^ was also employed for QTL analysis and validation (Supplementary Fig. 3). SubILs with shorter introgressions were obtained for chromosomes 3, 7 and 11 from the F_2_ populations of the crosses of the corresponding ILs with MM. DNAzol (Life Technologies, USA) was used to isolate DNA from the F_2_ populations and Single Nucleotide Polymorphisms (SNP) SOLCAP markers (available at https://solgenomics.net/) were employed to select the plants. Markers were resolved using High Resolution Melting analysis. Ailsa Craig wildtype (wt) (LA2838A) and the nearly isogenic Ailsa Craig *y*/*y* (LA3189) harboring the *colorless fruit epidermis* (*y*) mutation were also studied. Five plants per RIL, 10 plants per IL and subIL, and 20 plants of MM, Ailsa Craig wt and Ailsa Craig *y*/*y* were grown in a polyethylene greenhouse at the Estación Experimental La Mayora, CSIC, Spain.

### QTL analysis

The genotyping of the RIL population^45^ with the 8K SNP SOLCAP Infinium chip^46^ was employed to construct a linkage map containing 4885 SNP markers using JoinMap^®^ 4.0 software (Kyazma BV, Wageningen, the Netherlands) with the maximum likelihood algorithm and the Kosambi mapping function. QTL analysis was carried out with the software MapQTL^®^ 5.0 (Kyazma BV, Wageningen, the Netherlands) using interval mapping and subsequent QTL modeling by multiple QTL mapping after automatic cofactor selection. Permutation tests with 10,000 resamplings were employed to determine the probability associated to each QTL. A reduction of the map to 1302 informative SNP markers was performed for computational purposes to carry out a composite interval mapping^47^ and epistatic analysis using IciMapping^48^. QTL map was drawn with Mapchart 2.2 (ref. ^49^).

Any change in the amount of cuticle deposited (cuticle mass) also modifies the amount of cuticle components, unless their percentage distribution is modified. For this reason, the QTL analysis of the different cuticle components was carried out with the percentage and not the amount. Unless indicated otherwise, all the QTLs identified for the amount of cuticle also affected the amount of phenolics, waxes, cutin and polysaccharides.

### Gene expression analyses

Trizol^®^ Reagent (Life Technologies, USA) was used to isolate RNA from tomato fruit peels at different stages of development (immature green, mature green, breaker, and red ripe). Genomic DNA was removed with RNase-free DNase and RNA was cleaned with the Nucleospin RNA clean-up kit (Macherey-Nagel, Germany). First-strand cDNA synthesis was carried out with the Super Script III First-Strand Synthesis Super Mix for qRT-PCR (Invitrogen, USA). Relative transcript amount of the different genes was measured by RT-qPCR using SsoAdvanced™ SYBR^®^ Green Supermix (Bio-Rad, USA) following the ΔCt method with three control genes, *CAC*, *EXPRESSED*, and *SAND* previously employed for tomato peel analyses^24^. Three biological replicates corresponding to pools of epicarp pieces from different tomatoes and plants were analysed. For each biological replicate three technical replicates were performed. Primers used for amplification are shown in Supplementary Table 5. Comparison of the mRNA sequences of MM and TO-937 genes was carried out to identify changes that would result in non-conserved amino acid substitutions.

### Tissue sectioning and staining

Small pericarp pieces of three fruits per genotype, each fruit from a different plant, were fixed in a formaldehyde, acetic acid and ethanol solution (1:1:18), dehydrated in an ethanol dilution series (70–95%) and embedded in a commercial resin. Samples were cross-sectioned into slices 4 µm thick using a microtome (Leica, Germany) and stained with Sudan IV to visualize the cuticle. For the comparison of IL and subILs with MM, six fruits of MM, each from a different plant, were studied. Cuticle thickness was estimated from 30 measurements of cross-sectioned samples using the image analysis program Fiji. Ten non-seriated sections per biological replicate were analysed and 1–2 measures taken in each section. To avoid the effect of cutinized anticlinal cell walls or ridges, cuticle thickness was measured in the central region of cells, since this area remains constant throughout fruits. Cuticle area of 10 non-seriated cross-sections per biological replicate were calculated with the software Fiji and referred to the weight of the cuticle surface area to estimate cuticle density, following the protocol already established in^6^. Cuticle invagination was estimated from a minimum of 15 non-seriated cross-sections per biological replicate using an arbitrary index, as reported in^7^. This index accounts for how much the anticlinal and inner periclinal walls of epidermal and subepidermal cells are cutinized. 0 means no cutinization of the epidermal anticlinal cell walls; 0.25: the upper half of the epidermal anticlinal cell walls is cutinized; 0.5: epidermal anticlinal cell walls are completely cutinized; 0.75: additionally, half of the inner periclinal epidermal cell wall is also cutinized; 1: all the epidermal cell walls are cutinized. Values >1 indicate additional cell wall cutinization of the first hypodermal cell layer following the same criteria as for the epidermal cell layer.

### Cuticle isolation and cuticle component analyses

Cuticles were enzymatically isolated from tomato fruits following the protocol already stablished^6^ using an aqueous solution of sodium citrate with mixture of cellulase and pectinase and NaN_3_ to prevent microbial growth. Isolated cuticles were air dried and stored under dry conditions. Cuticle surface area was calculated using Fiji and 20 measures per genotype, 40 in the case of MM. Samples were then weighed in order to determine the mass of cuticle per unit surface area. Cuticular waxes were removed by heating at 60 °C 0.1 g of isolated cuticles in 100 mL chloroform: methanol (2:1 v:v) for 2 h. Cutin isolates were obtained after refluxing the dewaxed cuticles in a 6 M HCl aqueous solution for 12 h. Phenolic components were estimated after cutin depolymerization in a UV-VIS spectrophotometer (Pharmacia Biotech, NJ, USA) following a protocol previously described^50^. Percentages of cuticle components were estimated from five samples per genotype, except for MM where 10 samples were determined.

### Statistics

Data are expressed as means ± standard error (SE). *T* tests were used to compare the parental lines MM and TO-937, and Ailsa Craig wt and *y*/*y*. One-way ANOVA and Dunnett’s tests were used to compare the ILs or subILs with MM. Percentages were arcsin-transformed for statistical analyses, although they are presented untransformed. Pearson’s coefficient was employed to correlate the different variables studied. Asterisks indicate differences at *p < 0.05; **p < 0.01; ***p < 0.001.

## Supplementary information

Supplemental material

## Data Availability

The data that support the findings of this study are available from the corresponding author upon reasonable request.
