# Supplementary material for: Genome-wide QTL analysis of tomato fruit cuticle deposition and composition
Source: Hortic Res. 2021 May 1;8:113. doi: 10.1038/s41438-021-00548-5 (PMC8087829; doi:10.1038/s41438-021-00548-5)
Supplement: Supplementary file 1 — Supplemental material [file 41438_2021_548_MOESM1_ESM.docx]

**Genome-wide QTL analysis of tomato fruit cuticle deposition and composition**

Rida Barraj Barraj, Patricia Segado, Rocío Moreno-González, Antonio Heredia, Rafael Fernández-Muñoz, Eva Domínguez


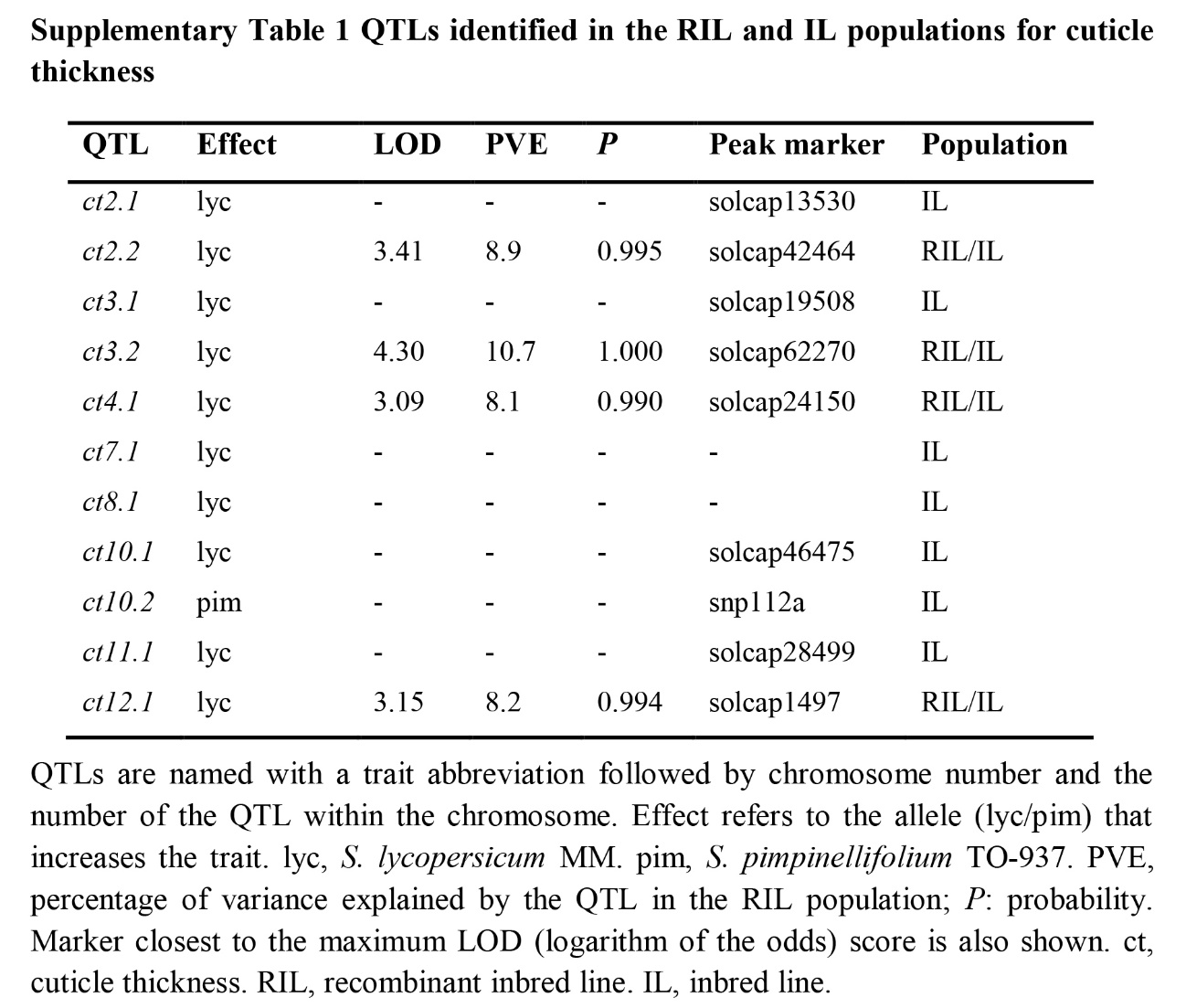


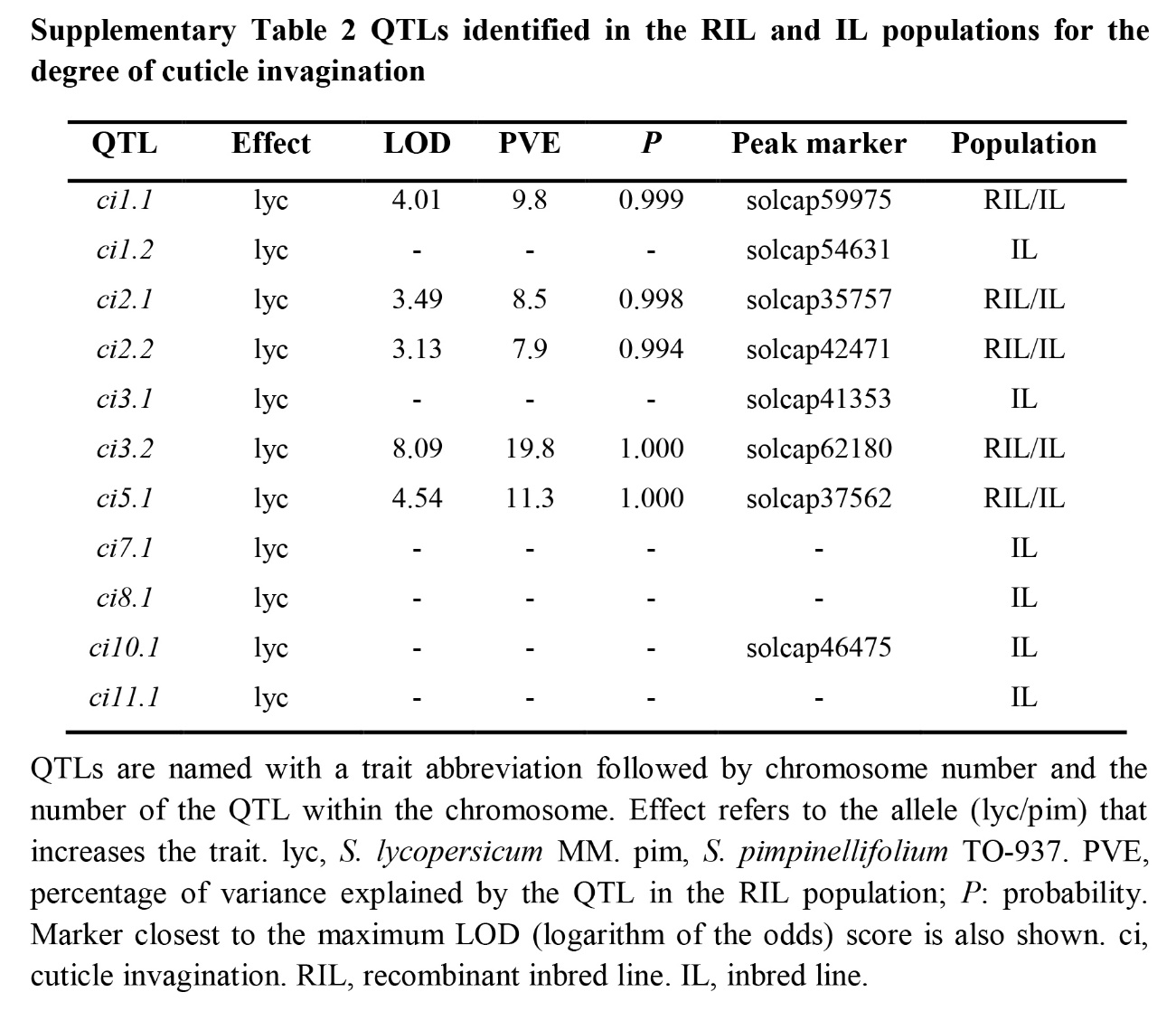


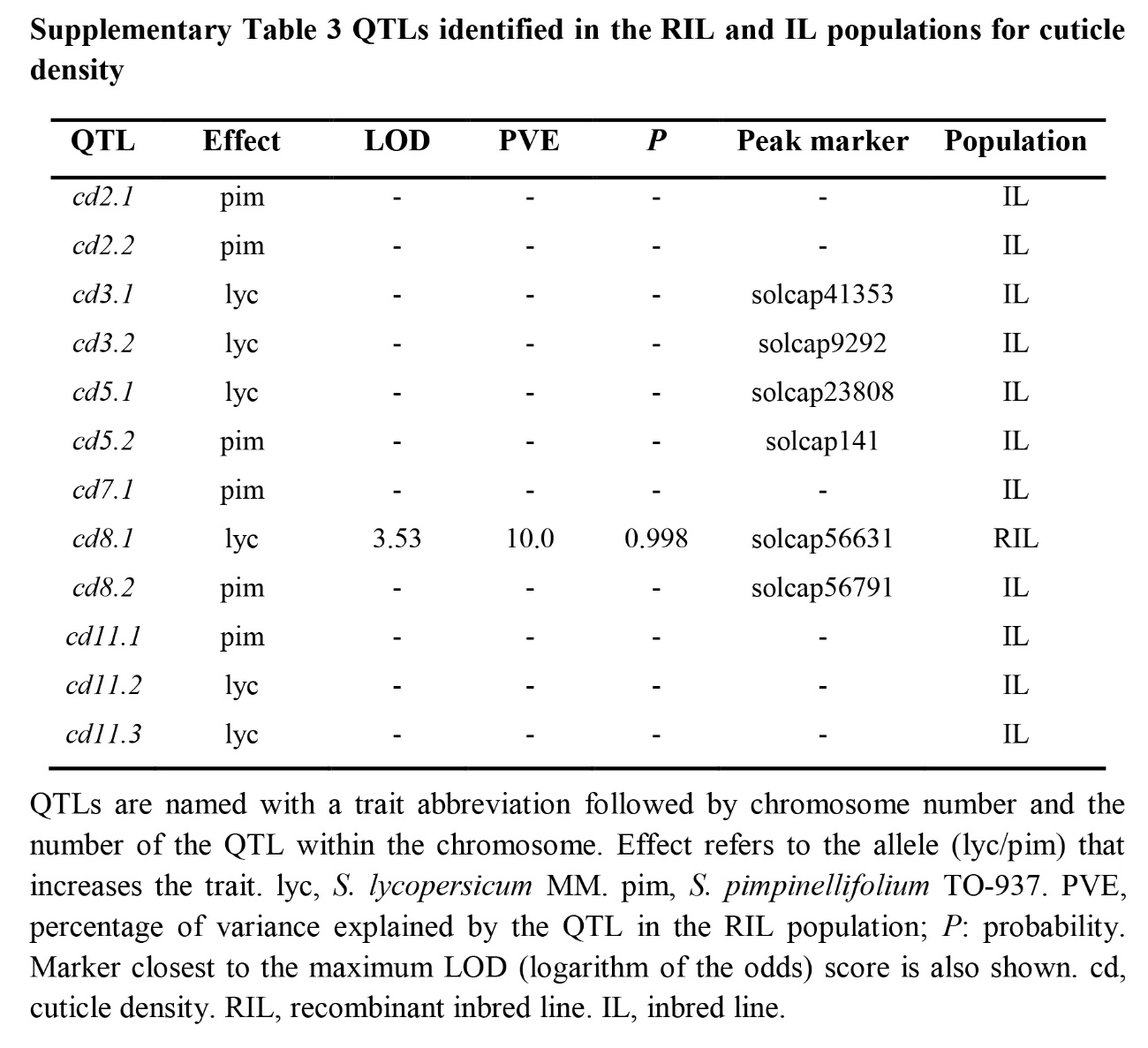


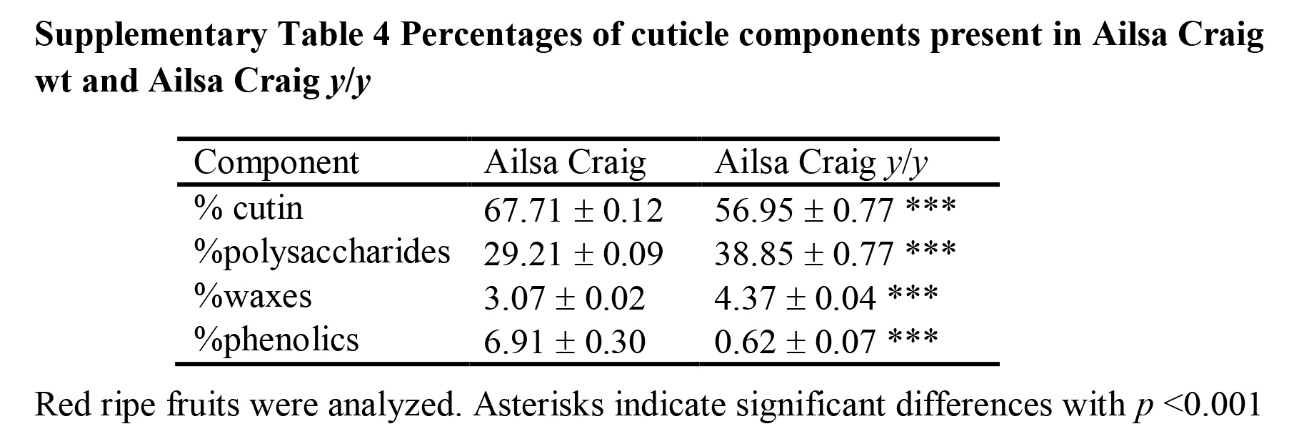


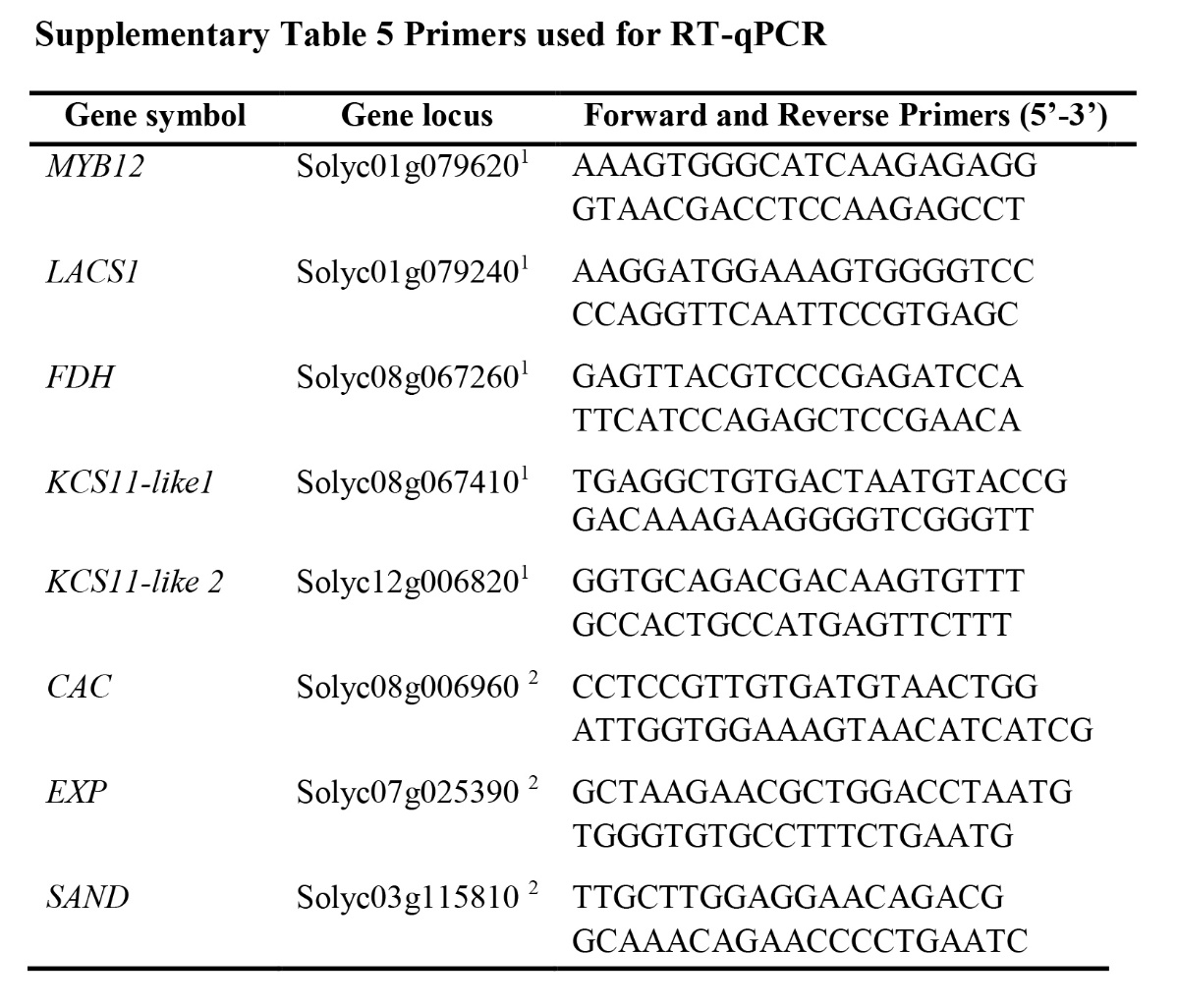


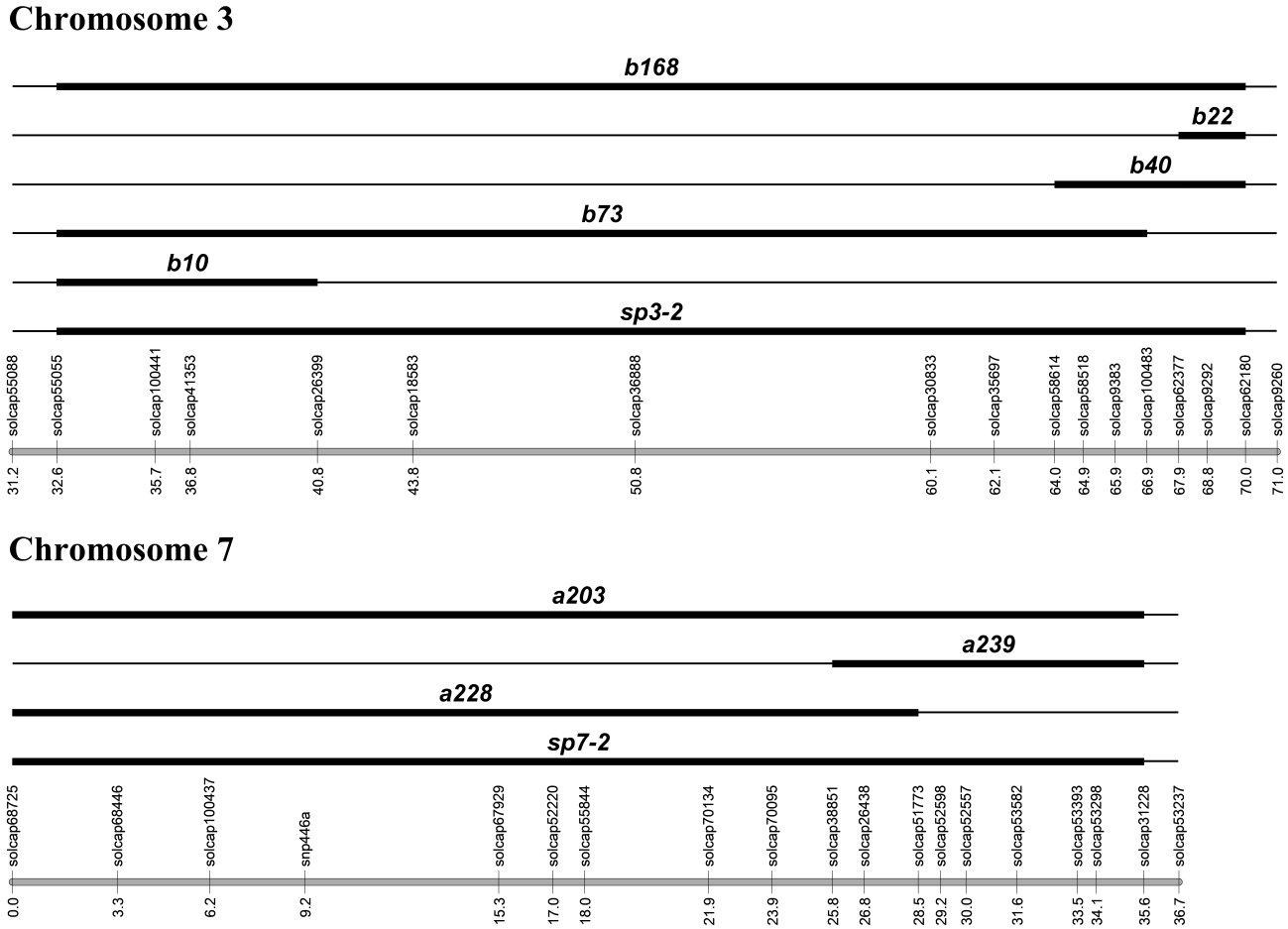


**Supplementary Fig. 1** **Schematics of the subILs for chromosomes 3 and 7.** Bold lines represent TO-937 genome. Inbred line (IL).


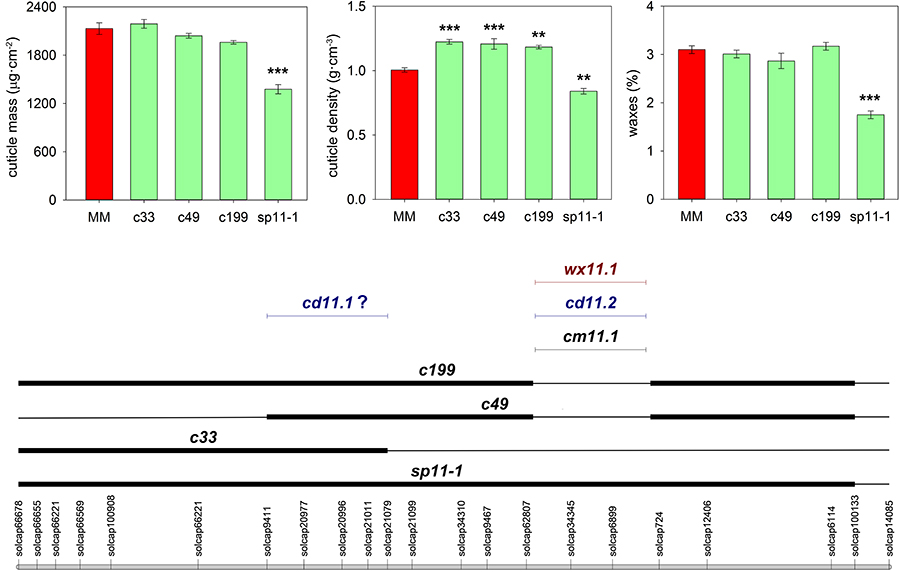


**Supplementary Fig. 2** **SubILs derived from IL sp11-1.** Top, cuticle mass, density and percentage of waxes for the subILs and sp11-1 compared to the parental line MM. Asterisks indicate significant differences with MM. Bottom, schematics of the subILs showing the first 80cM in the RIL map. Bold lines represent TO-937 genome. QTL regions for percentage of waxes (wx) cuticle mass (cm) and cuticle density (cd) are shown. c33, c49 and c199 subILs. Inbred line (IL).


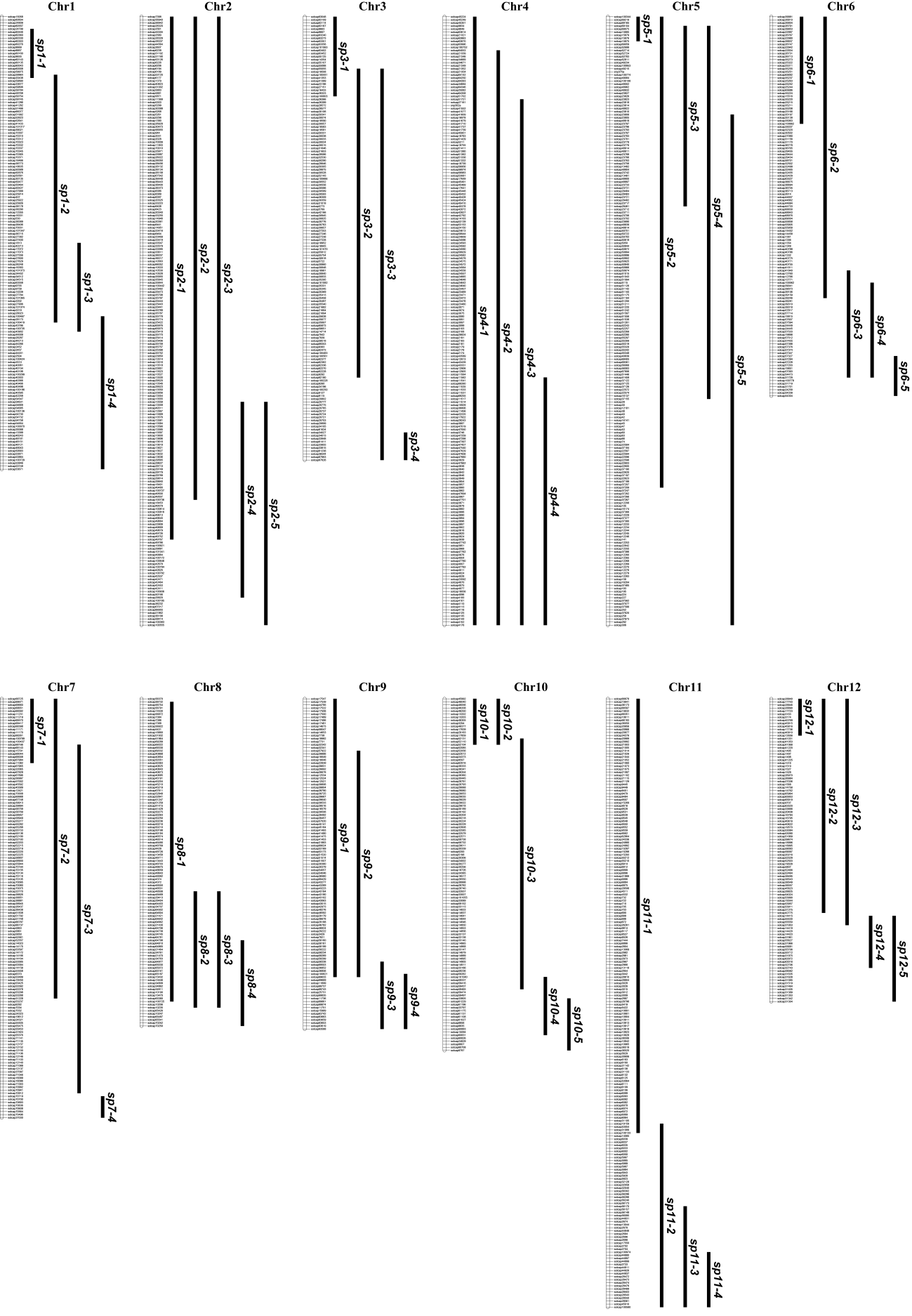


**Supplementary Fig. 3 Schematics of the sp ILs.** The TO-937 introgressions contained in each line are shown with bold lines. Chromosome (Chr). Inbred line (IL).
